# PyDSLRep: A domain-specific language for robotic simulation in V-Rep

**DOI:** 10.1371/journal.pone.0235271

**Published:** 2020-07-01

**Authors:** Andrés C. Jiménez, John P. Anzola, Vicente García-Díaz, Rubén González Crespo, Liping Zhao

**Affiliations:** 1 Department of Electronic Engineering, Los Libertadores Foundation University, Bogotá, Colombia; 2 Department of Computer Science, University of Oviedo, Asturias, Spain; 3 Department of Computer Science, International University of the Rioja, Avenida de la Paz, Logroño La Rioja, Spain; 4 Department of Computer Science, University of Manchester, Manchester, United Kingdom; IRHS, INRA, AGROCAMPUS-QUEST, Universite d’Angers, SFR 4207 QUASAV, FRANCE

## Abstract

Calculating forward and inverse kinematics for robotic agents is one of the most time-intensive tasks when controlling the robot movement in any environment. This calculation is then encoded to control the motors and validated in a simulator. The feedback produced by the simulation can be used to correct the code or to implement the code can be implemented directly in the robotic agent. However, the simulation process executes instructions that are not native to the robotic agents, extending development time or making it preferable to validate the code directly on the robot, which in some cases might result in severe damage to it. The use of Domain-Specific Languages help reduce development time in simulation tasks. These languages simplify code generation by describing tasks through an easy-to-understand language and free the user to use a framework or programming API directly for testing purposes. This article presents the language PyDSLRep, which is characterized by the connection and manipulation of movement in mobile robotic agents in the V-Rep simulation environment. This language is tested in three different environments by twenty people, against the framework given by V-Rep, demonstrating that PyDSLRep reduces the average development time by 45.22%, and the lines of code by 76.40% against the Python framework of V-Rep.

## Introduction

Robotic agents are widely used for industrial, surveillance, and search and rescue tasks. However, these applications have increased their development complexity in both hardware and software, as they rely on increasingly complex algorithms to perform them. This has moved developers to use techniques and tools that enable robotic agents to have more autonomy, depending on the environment in which they are deployed.

Currently, developers need to calculate and assess the development of a robotic agent, and this has a direct impact in the increase of development time, due the generation of a simulation model, which in several cases is independent of the software models implemented in physical agents, increasing the designer’s work.

The design of this type of tests is usually done using Domain-General Languages (DGLs) that extend APIs or high-level libraries for robotic applications development. However, these Domain-General Languages lack the required semantics or abstractions needed to develop movement actions, making it necessary to debug the code before the simulation stage [1].

There are languages capable of increasing the abstraction level and reducing development time by representing clearer semantics and allowing more simplicity in writing code. These languages, which rely on model generation and domain-specific concepts, are known as Domain-Specific Languages (DSLs). Domain-Specific Languages are becoming popular in robotics due to their flexibility, automation, and encapsulation of repetitive tasks, which results in a decrease of common errors in code generation. DSLs can also generate code that can be integrated easily to a GPL to handle API’s or libraries for specific use in platforms for real-world environments [2–5].

There are several tools that can be used to generate DSLs. These are divided into internal and external tools. Internal tools are extensions of GPLs like LUA or RUBY by adding new expressions or functionality. One example is the virtual simulation environment V-REP, which uses LUA to describe and control the environment and robotic agents being simulated [6].

External DSLs define their own syntax and concepts, which must be related to the desired application domain. For this type of DSLs, there are editors like Xtext, which allow the creation of a language grammar by designing its syntax and creating rules through a language called Xtend, which is used to implement grammar definitions, design the interpreter, and generate the DSL code [7].

Dejanovic et al. create a language based in Xtext called textX, to build a DSL in Python. This DSL allows to create Python code and presents an example of robotics application to simulate movements in a discrete environment. However, it does not demonstrate a direct application or the kinematic modeling of the robotic agent [1].

Currently, DSLs are oriented to manipulating robotic arms and describing the parts or robotic agents [8]. The goal of DSLs in the control of robotic arms in focused on generating movements to manipulate elements by allowing the modification of kinematic parameters. Conversely, DSLs such as the Universal Robotic Description Format (URDF) by ROS, allow describing all the parts of the robotic agents, specifying dynamic and kinematic parameters and allowing to model robotic agents for displacement in a real environment. However, it does not model the interaction of the robotic agent with obstacles in the environment [9].

This limitation prompted the development of an internal DSL called, to code movement tasks in robotic agents, and oriented to their validation using the V-REP simulation environment. This language offers a set of rules that abstract the kinematic behavior of the robot, helping reduce complexity in code development and testing by abstracting the functional and software architecture of the robotic agent, avoiding errors in the generation of the kinematic model. As it was developed using Xtext, it can be easily used in any operating system. Its text editor features auto-complete, highlights reserved words, and shows errors. Furthermore, generates the connection to V-REP’s proxy through a Python script, which can be easily modified by the user to embed it directly in a differential robotic platform, ensuring applicability and reusability in physical robotic agents.

To improve code generation, PyDSLRep divides code description into 4 stages. The first stage is modeling the differential robot, taking as a variable the size of the wheels to generate the model for forward and inverse dynamics. The second stage is declaring the robotic agent instances. The third is describing the movements that the robotic agents can perform, either serialized or concurrent, according to the user’s needs. Finally, the fourth stage groups the robotic agents in the environment and executes the movements.

The rest of the article is structured as follows. Section 2 presents the works performed in the implementation of DSLs oriented to robotic systems. Section 3 describes the kinematic model of the robotic agent modeled in PyDSLRepCode. The creation of the development environment is explained in Section 4. Section 5 presents the evaluation and discussion of the results obtained with, compared to the Python framework. Finally, Section 6 presents conclusions.

## Related works

Robotic agents have been employed for a wide variety of work domains, ranging from planning, mapping, locations, and assistance tasks. Several design architectures have been used for development in these work areas, with the goal of reducing software development time.

Silva et al. [10] establish a metamodel for the requirements analysis and software development for a generic system of robotic agents. This metamodel establishes a methodology based in software engineering, for the study of the agents’ requirements, by implementing different roles for the agents and how they have to share information. However, this metamodel only provides guidelines for roles and information specifications among different levels.

Like Silva, other authors have established frameworks and methodologies for the control [11–15], planning [16–18], and communications [19–21] of robotic agents, but they present the characteristics of DSLs to reduce development time in a flexible and efficient manner, and at the same time, increasing autonomy between hardware and software [8]. This autonomy is dependent on the domain knowledge for which the robotic agents are being used; this is, the problem requirements, proposed objectives, and system restrictions.

Klotzbücher et.al. [22] establishes an internal DSL based on LUA, based on the Model Driven Engineering (MDE) approach, to standardize the representation of Task Frame Formalism (TFF) for industrial robotic arms, by modeling the language through hierarchical definitions and orienting the user intuitively, without direct influence of hardware during program design. This is achieved by implementing the design flow through DSL representation, according to the four levels of standardization of the OMG group, applying the MDE approach. In this case, the level M0 is in charge of manipulating the robotic arm. Although software design in the previous case is not directly related to hardware, it depends on pre-existing frameworks or libraries for manipulating robotic arms [23].

Frigerio et al. [22] present a DSL focused on reducing the calculation of transformation matrices required to control actuators in robotic arms, by generating models to be implemented according to the kinematic parameters of the robotic arm. This DSL develops code generation for implementation in positioning tasks for a robotic arm inside a simulation, emphasizing the use of models to be reused in physical robots. However, this is only possible in agents that have the same kinematics.

The previous works present DSLs oriented to application in robotic arms, which prevents them to be used in other types of mobile platforms such as differential or wheeled robots. Loetzsch et al. [24] propose a DSL called XABSL, for implementation in autonomous agents in complex environments. XABSL minimizes the developer’s role in code creation by using finite state machines to represent the actions to be executed by the robotic agent. The language presented by Loetzsch has been used in RoboCup, showing good results in the team that implemented it. However, robotic platforms must have a framework compatible with the language, which prevents implementation in proprietary platforms. Furthermore, it cannot model the entire agent, as it only provides selection mechanisms by action, and it does not allow the possibility of performing simulation tests prior to implementation.

Similar to Loetzsch, Campusano et al. [25] design a DSL that represents code functionality by finite state machines, but in this case, they can take four possible states, as chosen by the user. It also allows the programmer to see the solution deployment in real-time by using live programming, which reduces programming time by allowing code debugging without the need for compilation or sending the code to a test unit. Conversely, the language can generate code to create communication nodes with the Robotic Operating System (ROS) and use the implemented algorithms directly. The drawback of this language is that it avoids evaluating proprietary algorithms and, like XABSL, it is not compatible with simulators for testing the generated program.

DSLs have also been oriented to graphical programming. In these cases, the user programs using blocks that contain pre-programmed routines. Pot et al. [26] developed Choregraphe, a development environment that includes the characteristics presented by Campusano by showing the results of the programs developed by users in real-time and inside a virtual environment. Each of the blocks contained in Choregraphe can be modified through a Python script, allowing the user to implement their own algorithms or libraries for remote information processing. However, as this software is dedicated to NAO humanoid robots, it cannot be used for programming other robotic agents.

Another advantage of Choregraphe is the use of Python scripts. Blasco et al. [27] highlight the importance of using this high-level language to control robotic agents in high-complexity tasks. In contrast with the previous works mentioned, PyDSLRepCode is a language oriented to movement control in mobile robotic agents. This language features a simple user syntax, simplifying the connection and direct verification with V-REP before direct implementation in the test agent. Another great advantage of PyDSLRepCode is the generation of organized code in Python, which allows the user to reuse the code to add the robotic agent’s API for direct motor manipulation or to implement navigation algorithms directly.

## PyDSLRepCode kinematic description

Every movement of a robotic agent is represented by its kinematic description, which describes the position in which the robot can be according to the speed of its wheels (forward kinematics) or the speed that every wheel should have to achieve the desired orientation and position (inverse kinematics.) In every case in which the developers wish to control the movement of the robotic agent, they must calculate the forward and inverse kinematics of the robotic agents to generate the software code to manipulate the agent’s motors. Errors might be introduced during the execution of this task, resulting in the execution of movements with errors by the robotic agents and preventing the completion of the desired navigational tasks.

PyDSLRepCode simplifies writing code to control the robotic agent’s movements. This language embeds the kinematic description of the differential robotic agent, as this is one of the most commonly used structures for navigational tasks [28]. To achieve this, PyDSLRepCode describes code development in four stages: 1) Describes the robotic agent components to calculate forward kinematics. 2) Instances the robotic agents. 3) Assigns to each agent the movement actions required by the developer (inverse kinematics.) 4) Creates the environment to contain the robotic agents and assigns the execution order of the movements developed in stage 2.

Down below is a description of each of PyDSLRepCode’s coding stages, demonstrating the abstraction capability to describe movement in differential robotic agents.

### Direct kinematic in PyDSLRepCode

Differential robots are characterized by having two wheels, which are placed parallel to each other along the same axis and separated from each other by a distance d. Each of the wheels has an autonomy of movement represented by a velocity vector ***v***_***w***_. This allows controlling the rotational movement of the robot. As both wheels are placed along the same axis, the rotational movement has a common rotation point for both wheels, which is where the agent presents rotation. This point is known as Instantaneous Center of Rotation (ICR).

**Fig 1:**
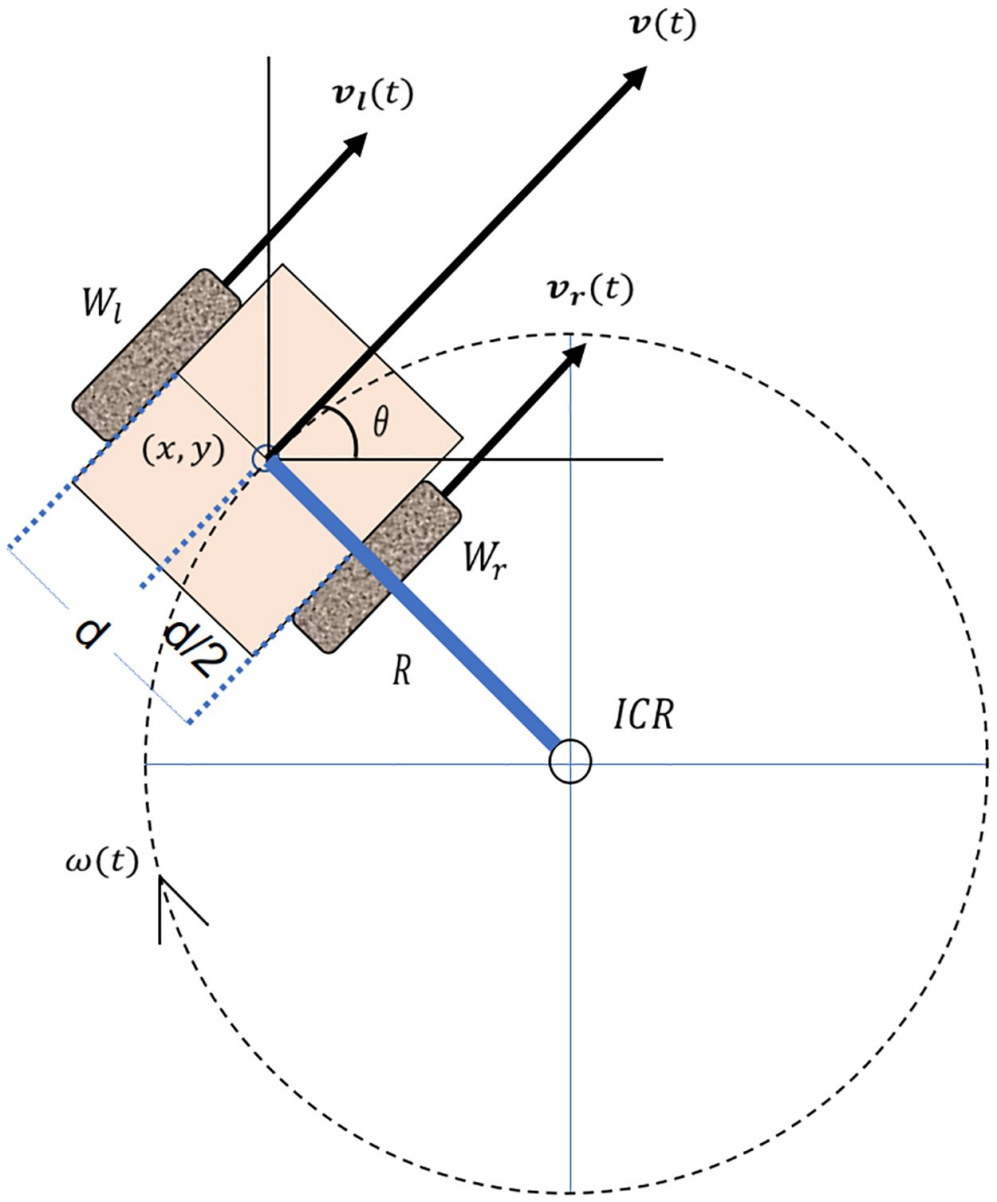
Kinematic representation of a two-wheeled differential drive robot.

Fig 1 shows the action of velocity vectors in each of the wheels, with respect to the ICR. Each of these velocities has a direct effect on the trajectory of the robotic agent, generating the same angular velocity *ω*(*t*) in both wheels with respect to the ICR. Eq (1) presents the calculation of velocities in the wheels, according to the curvature radius *R* with respect to the ICR point.

$$\begin{array}{c} v_{r,l}=\omega \left(t\right)(R\pm \frac{d}{2}) \end{array}$$ (1)

From Eq (1), we can clear the curvature radius R and the angular velocity *ω*(*t*) generated by the rotational movement, obtaining Eqs (2) and (3).

$$R=\frac{d(v_{l}\left(t\right)+v_{r}\left(t\right))}{2(v_{r}\left(t\right)-v_{l}\left(t\right))}$$ (2)

$$\begin{array}{c} \omega \left(t\right)=\frac{v_{r}\left(t\right)-v_{l}\left(t\right)}{d} \end{array}$$ (3)

**Fig 2:**
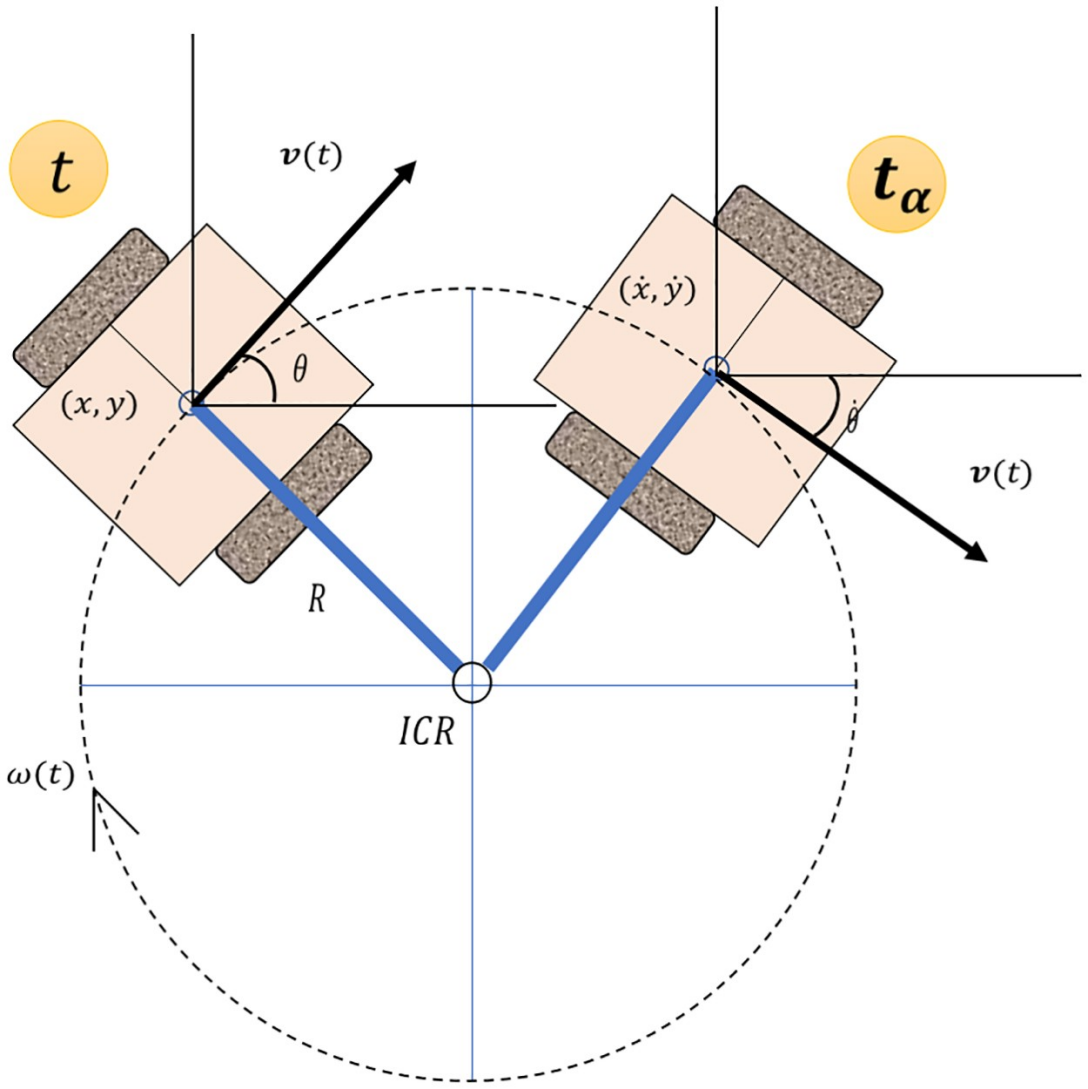
Direct kinematic movement after *t*_*α*_ time.

Using trigonometric properties, and assuming that the point d/2 is located at the position (*x*, *y*) with an angle *θ* respect the x-axis, we obtain the position *ICR* = [*x* − *Rsin*(*θ*), *y* + *Rcos*(*θ*)]. Taking this into account, if the robot moves at a speed *ω*(*t*) for a time *t*_*α*_ (Fig 2), its new position can be calculated with Eq (4).

$$\begin{array}{c} \left[\begin{array}{c} \dot{x}\\ \dot{y}\\ \dot{\theta } \end{array}]=[\begin{array}{ccc} cos(\theta_{t\alpha }) & -sin(\theta_{t\alpha }) & 0\\ sin(\theta_{t\alpha }) & cos(\theta_{t\alpha }) & 0\\ 0 & 0 & 1 \end{array}][\begin{array}{c} Rsin(\theta )\\ -Rcos(\theta )\\ \theta \end{array}]+[\begin{array}{c} x-Rsin(\theta )\\ y+Rcos(\theta )\\ \theta_{t\alpha } \end{array}\right] \end{array}$$ (4)

As is shown in Eq (4), both the angular velocity *ω*(*t*), the position of the robot in the Cartesian plane, and the reference point in the rotational movement ICR, depend directly on the velocity of each wheel and on the time in which energy is applied to the motors. Using this information, PyDSLRepCode builds a model of the robotic agent by asking the developer the wheels included in the robot model, in which the input of the radius and the port number through which communication will be made are mandatory.

**Fig 3:**
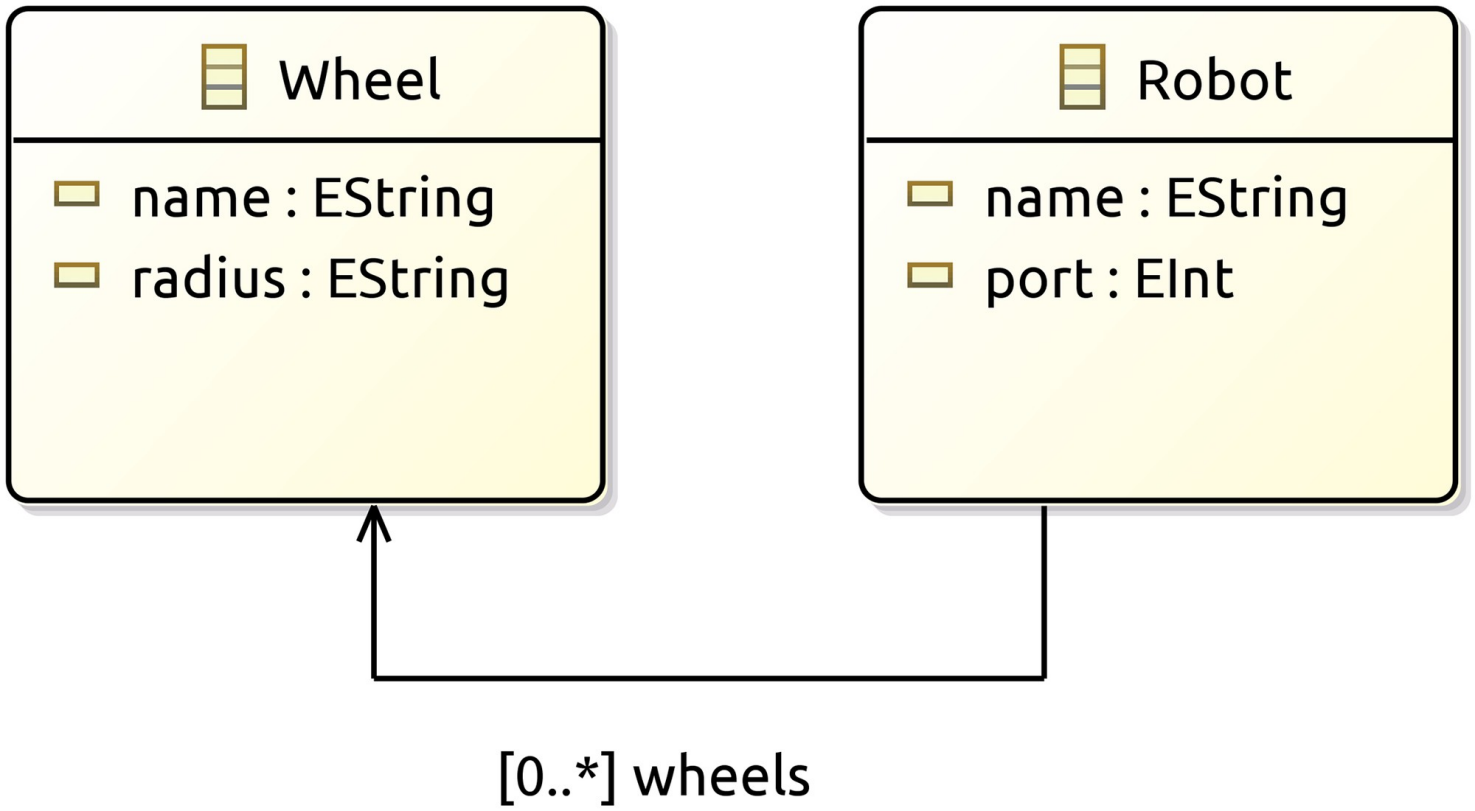
Class diagram direct kinematic behaviour PyDSLRepCode.

To identify each wheel and the robotic agents using them, PyDSLRepCode requires each wheel to have a unique identifier, which is represented by a ***string*** (Fig 3).

Another important aspect of PyDSLRepCode is that if the user characterizes more than one robotic agent, and these agents share the same wheel type, PyDSLRepCode allows the creation of a single instance of the wheel, which can be assigned to all the agents.

The abstraction power of PyDSLRepCode exempts developers from representing Eqs (1)–(4), taking the value d of separation between wheels directly from the references acquired through V-Rep, and effectively reducing the number of variables the user is required to encode.

### Inverse kinematic in PyDSLRepCode

By knowing the velocity of a robotic agent, its position can be calculated after a time *t*_*α*_, as shown in Eq (4). However, to move to a location or configuration in the environment (*x*, *y*, *θ*), the velocity and orientation of the robotic agent must be controlled. This problem can be solved by breaking down the velocity vector ***v***(***t***) in (*x*, *y*), integrating it with respect to time *t* (Eq (5).) From this, to simplify the displacement calculations and avoiding the problem of perpendicular displacement in differential robots, the speeds of the wheels are equalized by generating the movement matrix of Eq (6), where *ϕ* is the desired angle of rotation.

$$\begin{array}{c} \begin{array}{cc} x(t) & =\frac{1}{2}\int_{0}^{t}(v_{r}\left(t\right)+v_{l}\left(t\right))cos\left(\theta t\right)dt\\ y(t) & =\frac{1}{2}\int_{0}^{t}(v_{r}\left(t\right)+v_{l}\left(t\right))sin\left(\theta t\right)dt\\ \theta (t) & =\int_{0}^{t}\omega \left(t\right)dt \end{array} \end{array}$$ (5)

$$\begin{array}{c} \left[\begin{array}{c} \dot{x}\\ \dot{y}\\ \dot{\theta } \end{array}]=[\begin{array}{cc} cos(\theta ) & 0\\ sin(\theta ) & 0\\ 0 & 1 \end{array}][\begin{array}{c} \vec{r}\\ \theta \end{array}]+[\begin{array}{c} x\\ y\\ \phi \end{array}\right] \end{array}$$ (6)

**Fig 4:**
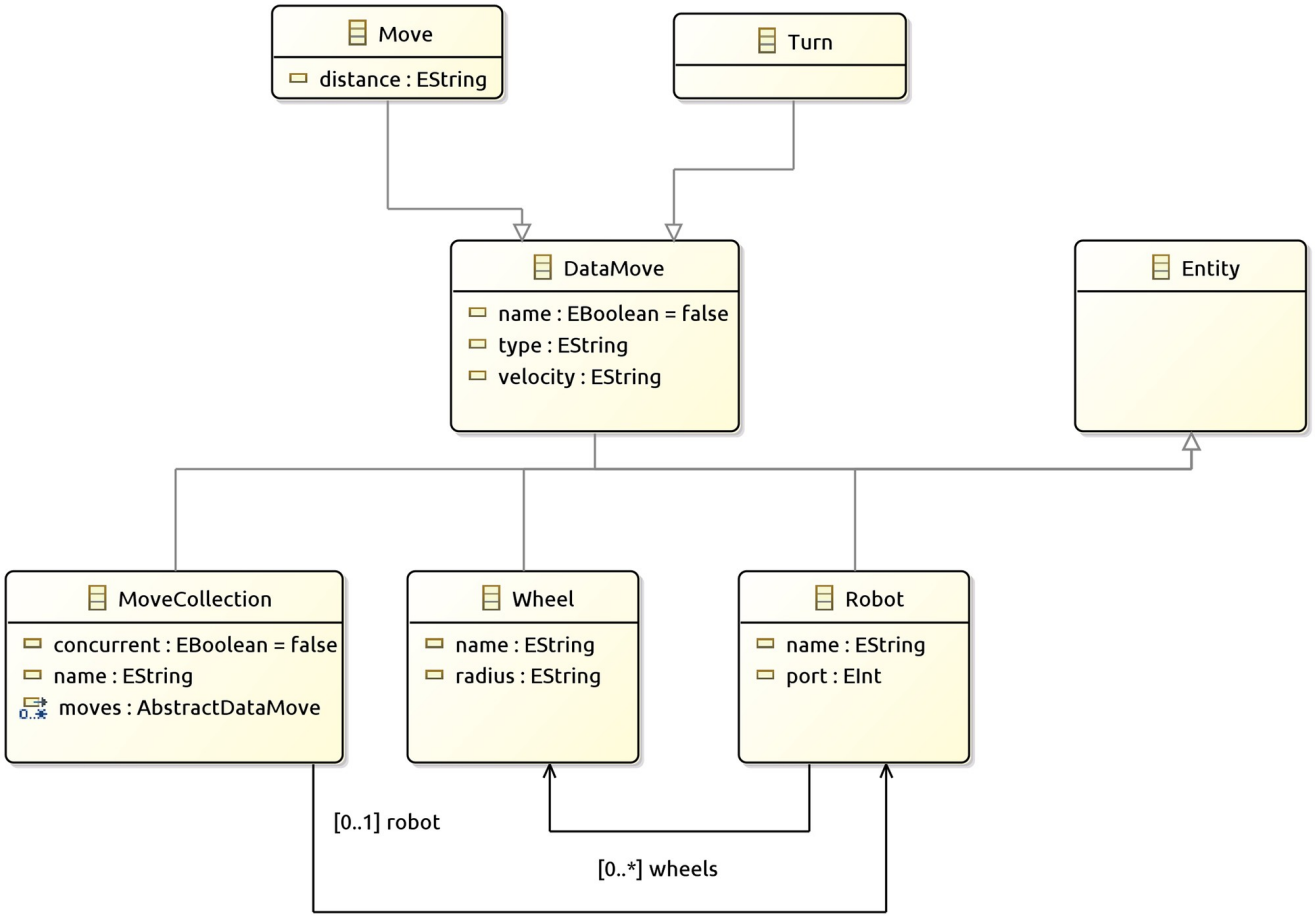
Class diagram inverse kinematic behaviour PyDSLRep.

To save the designer from having the calculate the integrals (5) and the calculation of velocities in the matrix (6), PyDSLRep groups the movement actions in a way that the user is only required to input the displacement distance and the velocity for the movement of the robotic agent, separating the movement actions in straight lines and turns, and thus avoiding the displacement problems in horizontal agents (Fig 4) During the input of information to instantiate a collection of movements, PyDSLRep requests two parameters. The first parameter is the assignment of a unique name, and the second is that the collection is related to a single robotic agent. This is to ensure the execution of actions by the robotic agent at the desired moment.

**Fig 5:**
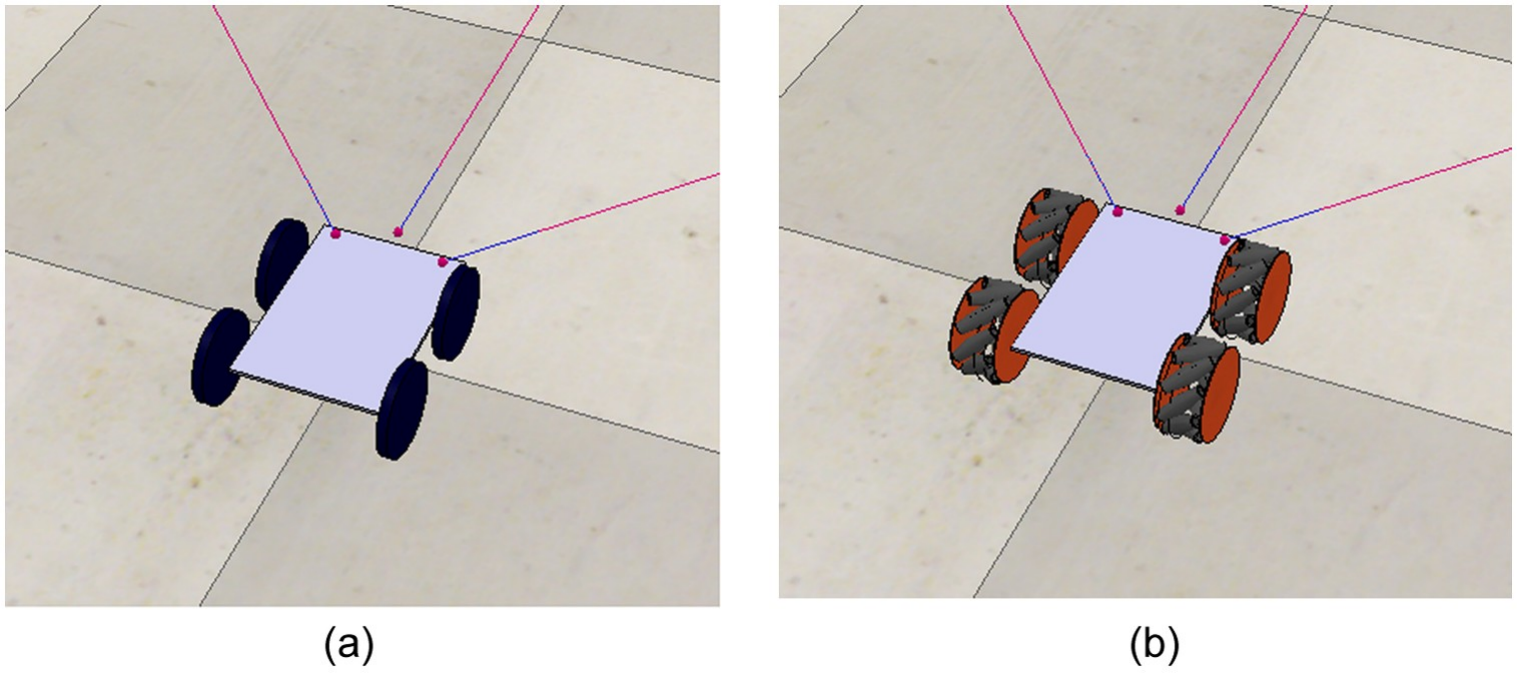
Four wheeled robots implemented in PyDSLRep (**a**) non holonomic (**b**) holonomic using mecanum wheels.

In addition to the differential model presented above, PyDSLRep also includes two additional models. The first one is a four-wheel robot Fig 5a, n which the calculations for inverse and direct kinematics are based on the article by Maulana *et.al*. [29]. While the second model is also four-wheeled, the motion of its wheels is omnidirectional or holonomic, which is possible when using mecanum wheels Fig 5b, The inverse and direct kinematics of this second model are based on the work of Zimmermann *et.al*. [30].

## Creation of the development environment

**Fig 6:**
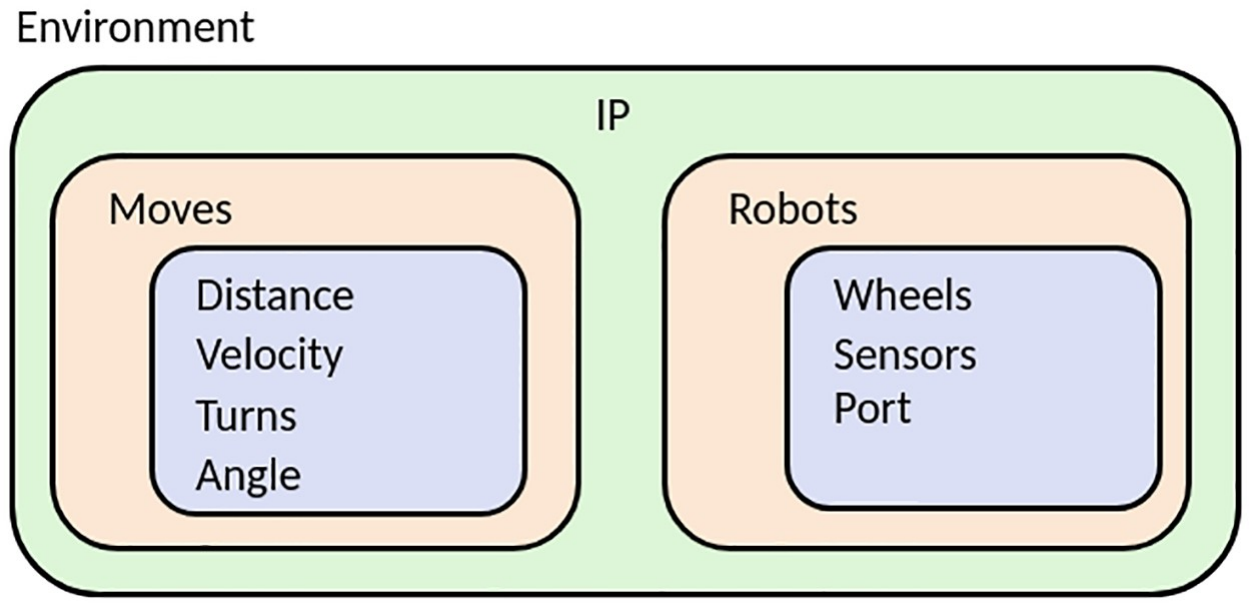
Code architecture of an environment.

The work environment in PyDSLRep contains the robotic agents’ instances and the collection of movements they will perform. This is possible by starting the development of each of the components of the robotic system. The creation process of the environment is done in four steps, which are described as follows. The steps follow the bottom-up architecture pattern, as shown in Fig 6:

1. Instantiating the components of the wheels. This requires the creation of at least two of these components. If necessary, as is the case if the robotic agent will connect to a remote host, an additional IP component is created, containing its respective IPv4 address.
2. Declaring and instantiating the robotic agents, which must contain the wheels, to generate their kinematic model.
3. Creating the collection of movements for each of the robotic agents that were instantiated.
4. Finally, creating the environment component, which contains the robotic agents and the movements desired, which were instantiated in previous stages.

The grammar created in PyDSLRep is shown in Table 1. This is used to instantiate components and declare instructions to complement the usability for each of them.

**Table 1:** Grammar implemented in PyDSLRep.

Keyword	Feature	Grammar rule
wheel	Creates a wheel component	***wheel*** wheel_name ***with radius*** (float in cm)
wheelH	Creates an holonomic wheel component	***wheelH*** wheel_name ***with radius*** (float in cm)
robot	Creates a robot component	***robot type_declaration*** robot_name ***with port*** (int) ***has*** {wheel_name_*L*_*x*_ wheelR_name_*R*_*x*_}***wheels with sensors*** {sensor_name_1 … sensor_name_5}
type two_wheels	Creates a two wheels differential robot component	***type*** tw
type four_wheels	Creates a four wheels robot component	***type*** fw
type four_wheelsH	Creates a four wheels holonomic robot component	***type*** fwh
sensor	Declares a distance sensor	***sensor*** senor_name
movement	Creates a collection of movements of a specific robot	***movement*** mov_name ***of*** robot_name {***move*** (float) ***meters*** at (float) ***turn right*** (int)}
parallel movement	Creates a collection of movements that are concurrent	***parallel movement*** mov_name ***of*** robot_name {***move*** (float) ***meters at*** (float) ***turn right*** (int)}
slam	Declares a movement to create a map	***slam***
environment	Creates a collection of robots with a specific collection of movements	***environment*** area_name ***has*** {robot_name_1 … robot_name_n} ***robots with*** {mov_name_1 … mov_name_n} ***moves***
ip	Creates an IP component as IPv4	***ip*** ip_name (int). (int). (int). (int)
move	Creates a linear movement component with a specific velocity	***move*** (float) ***distance_ declaration at*** (float)
turn	Creates a rotatory movement component	***turn rotatory_declaration***
left	Declares the orientation of the turn component 90 degrees to its left if there is not an ***angle_declaration***	***turn right*** (int)
right	Declares the orientation of the turn component 90 degrees to its right f there is not an ***angle_declaration***	***turn left*** (int)
turn until	Creates a rotatory movement component	***turn rotatory_declaration until*** {sensor_name}
port	Declares the port number of each robotic agent	***port*** (int)
meters	Declares the linear distance traveled in meters	***move*** (float) ***meters at*** (float)
centimeters	Declares the linear distance traveled in centimeters.	***move*** (float) ***centimeters at*** (float)

**Fig 7:**
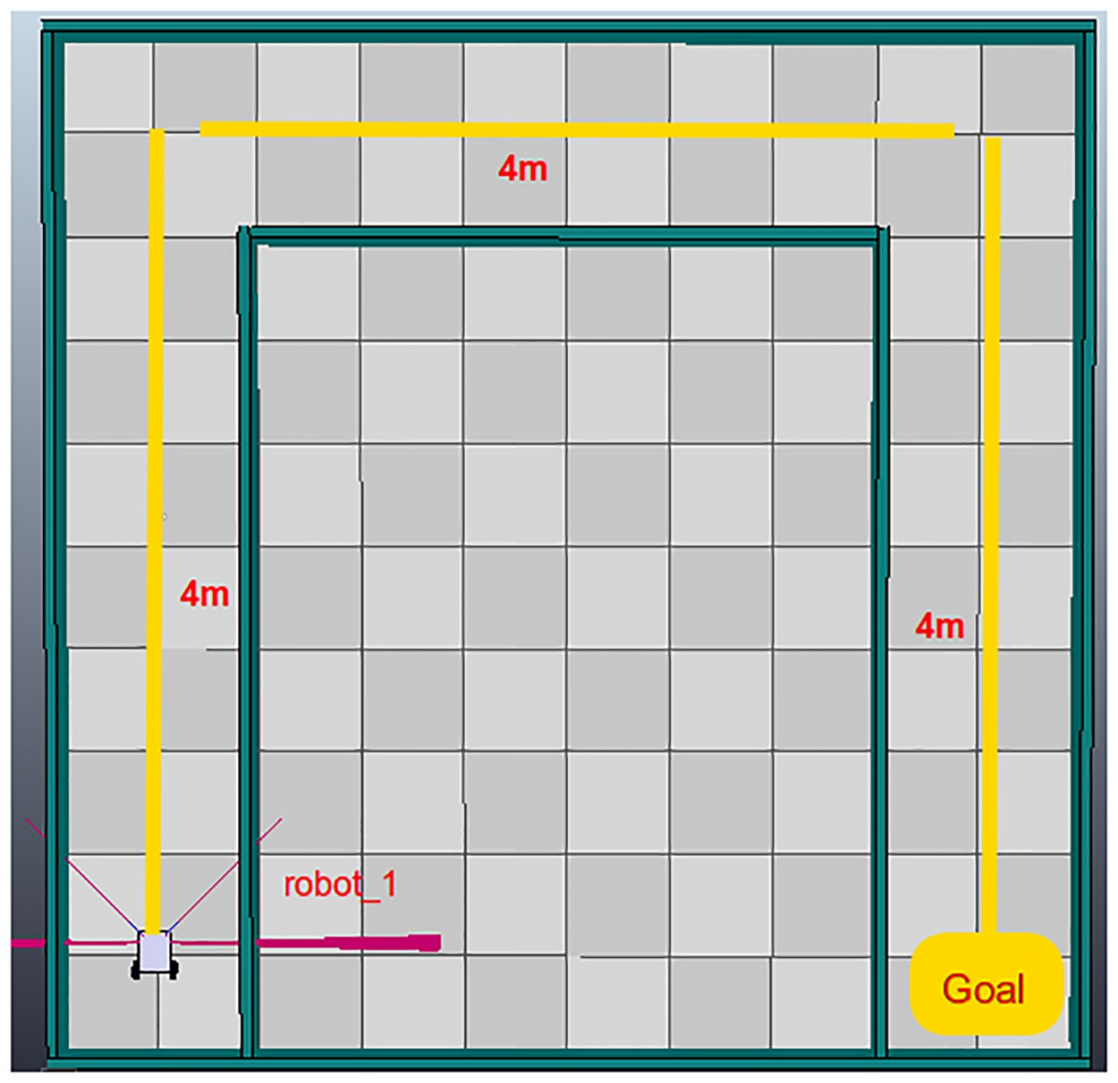
First basic environment created in V-Rep.

Fig 7 shows a differential robotic two-wheel agent that must travel a total distance of 12 meters to reach its final objective. To model this task in PyDSLRep, the grammar rules of Table 1 are applied, with the bottom-up design architecture, as shown in Table 2.

**Table 2:** Bottom-Up architecture design in PyDSLRep two wheel differential robot.

Step	Code
1	***wheel*** wheelR ***with radius*** 4.5
	***wheel*** wheelL ***with radius*** 4.5
2	***robot*** robot_1 ***type*** tw ***with port***
	19999 ***has*** {
	wheelL
	wheelR }***wheels***
3	***movement*** mov_1 ***of*** robot_1 {
	***move*** 4.0 ***meters***
	***turn right***
	***move*** 4.0 ***meters***
	***turn right***
	***move*** 400.0 ***centimeters*** }
4	***environment*** area_1 ***has*** {
	robot_1 } ***robots with*** {
	mov_1 }***moves***

Table 3 shows the the bottom-up architecture for a four wheeled robotic agent, meanwhile, Table 4 shows the implementation using an holonomic four wheeled robotic agent in the same environment Fig 7 Same as the differential robotic agent.

**Table 3:** Bottom-Up architecture design in PyDSLRep four wheeled robot.

Step	Code
1	***wheel*** wheelR0 ***with radius*** 4.5
	***wheel*** wheelR1 ***with radius*** 4.5
	***wheel*** wheelL0 ***with radius*** 4.5
	***wheel*** wheelL1 ***with radius*** 4.5
2	***robot*** robot_1 ***type*** fw ***with port***
	19999 ***has*** {
	wheelL0 wheelL1
	wheelR0 wheelR1}***wheels***
3	***movement*** mov_1 ***of*** robot_1 {
	***move*** 4.0 ***meters***
	***turn right***
	***move*** 4.0 ***meters***
	***turn right***
	***move*** 400.0 ***centimeters*** }
4	***environment*** area_1 ***has*** {
	robot_1 } ***robots with*** {
	mov_1 }***moves***

**Table 4:** Bottom-Up architecture design in PyDSLRep holonomic four wheeled robot.

Step	Code
1	***wheelH*** wheelR0 ***with radius*** 4.5
	***wheelH*** wheelR1 ***with radius*** 4.5
	***wheelH*** wheelL0 ***with radius*** 4.5
	***wheelH*** wheelL1 ***with radius*** 4.5
2	***robot*** robot_1 ***type*** fw ***with port***
	19999 ***has*** {
	wheelL0 wheelL1
	wheelR0 wheelR1}***wheels***
3	***movement*** mov_1 ***of*** robot_1 {
	***move*** 4.0 ***meters***
	***turn right***
	***move*** 4.0 ***meters***
	***turn right***
	***move*** 4.0 ***meters***
	***turn right*** }
4	***environment*** area_1 ***has*** {
	robot_1 } ***robots with*** {
	mov_1 }***moves***

**Fig 8:**
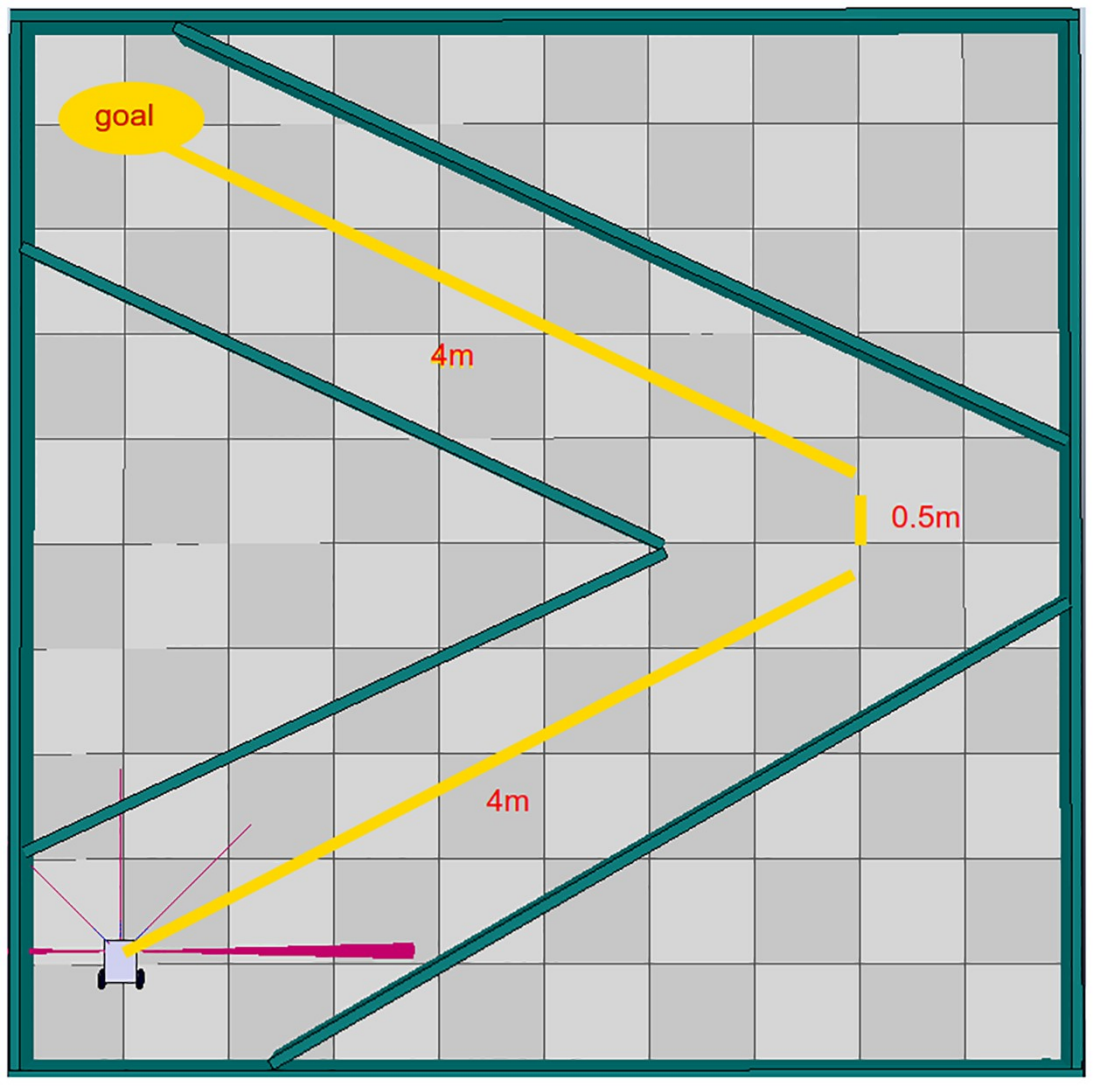
Second basic environment created in V-Rep.

In case the robotic agent needs to make turns at angles different than 90 degrees, as shown in Fig 8, turn commands must be implemented, specifying the direction and angle of rotation. Table 5 shows the development of the path using a two-wheel differential robotic agent. Four-wheel robotic agents are also capable of executing these motions.

**Table 5:** Bottom-Up architecture design in PyDSLRep two wheel differential robot third environment.

Step	Code
1	***wheel*** wheelR ***with radius*** 4.5
	***wheel*** wheelL ***with radius*** 4.5
2	***robot*** robot_1 ***type*** fw ***with port***
	19999 ***has*** {
	wheelL0 wheelL1
	wheelR0 wheelR1}***wheels***
3	***movement*** mov_1 ***of*** robot_1 {
	***turn right*** 45
	***move*** 4.0 ***meters***
	***turn left***
	***move*** 0.5 ***meters***
	***turn left*** 45
	***move*** 4.0 ***meters***
	}
4	***environment*** area_1 ***has*** {
	robot_1 } ***robots with*** {
	mov_1 }***moves***

PyDSLRep supports distance sensors, which can be used directly by robotic agents to generate motion autonomously. Table 6 shows the results of using a robotic agent with three frontal distance sensors, which are used to determine how much of the path should the agent rotate autonomously to continue with the following motion instruction.

**Table 6:** Bottom-Up architecture design in PyDSLRep two wheel differential robot with sensors.

Step	Code
1	***wheel*** wheelR ***with radius*** 4.5
	***wheel*** wheelL ***with radius*** 4.5
2	***sensor*** fSensor
	***sensor*** lSensor
	***sensor*** rSensor
3	***robot*** robot_1 ***type*** tw ***with port***
	19999 ***has*** {
	wheelL
	wheelR }***wheels with sensors***
	{fSensor, lSensor, rSensor}
4	***movement*** mov_1 ***of*** robot_1 {
	***move*** 4.0 ***meters***
	***turn right until*** {fSensor, rSensor}
	***move*** 4.0 ***meters***
	***turn right until*** {fSensor, rSensor}
	***move*** 400.0 ***centimeters*** }
5	***environment*** area_1 ***has*** {
	robot_1 } ***robots with*** {
	mov_1 }***moves***

**Fig 9:**
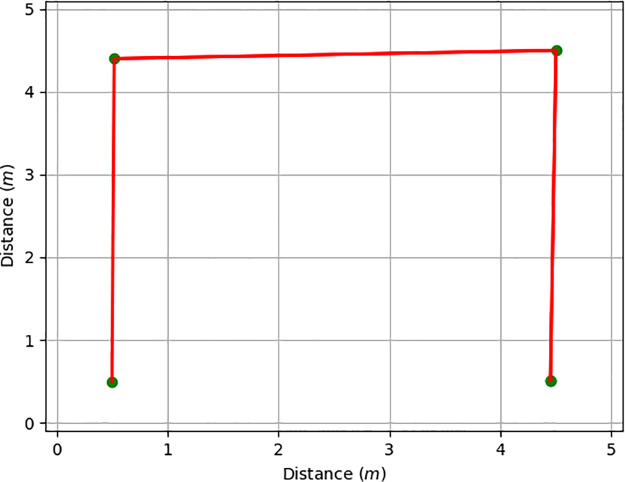
Topological map of the first test environment.

Finally, PyDSLRep contains an instruction for generating a topological SLAM, as demonstrated in *et.al*. [31]. For its use, the robotic agent needs to be equipped with at least five distance sensors, as shown in Fig 7. The generation of the map requires that the environment be closed. Table 7 shows the bottom-up architecture implemented for map generation. The generated map result is shown in Fig 9.

**Table 7:** Bottom-Up SLAM architecture design in PyDSLRep.

Step	Code
1	***wheel*** wheelR ***with radius*** 4.5
	***wheel*** wheelL ***with radius*** 4.5
2	***sensor*** fSensor0
	***sensor*** fSensor1
	***sensor*** fSensor2
	***sensor*** lSensor
	***sensor*** rSensor
3	***robot*** robot_1 ***type*** tw ***with port***
	19999 ***has*** {
	wheelL
	wheelR }***wheels with sensors***
	{fSensor0,fSensor1,fSensor2,
	lSensor, rSensor}
4	***movement*** mov_1 ***of*** robot_1 { ***slam*** }
5	***environment*** area_1 ***has*** {
	robot_1 } ***robots with*** {
	mov_1 }***moves***

## Evaluation and discussion

It is necessary to propose testing environments to assess PyDSLRep’s usability as compared to the native framework used for the simulation of robotic agents in V-Rep. To this end, this section presents two stages for the testing process. The first stage presents the methodology for conducting experiments using PyDSLRep and the Python framework. The second presents the results obtained and a discussion of the results. The PyDSLRep repository can be found in [32].

### Methodology

To validate the usability of PyDSLRep and the Python framework in the V-Rep simulator, three scenarios are introduced. The first two scenarios contains obstacles with robotic agents and two groups of users with knowledge of the Python programming language. The differentiating factor of the first group against the second group is the lack of knowledge about the Python framework for V-Rep. The third scenario is a non uniform environment, with obstacles with different shapes.

**Fig 10:**
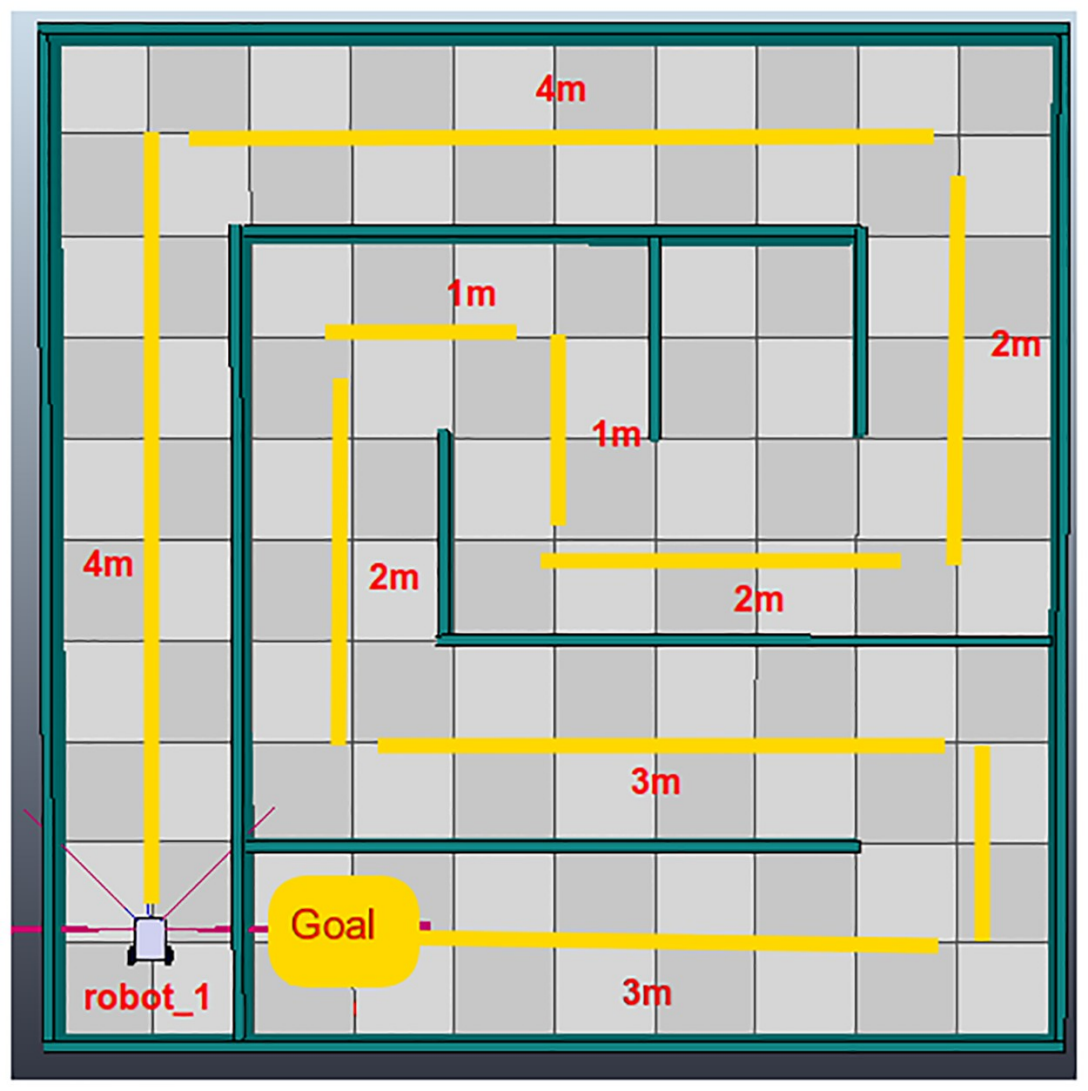
First test environment created in V-Rep.

**Fig 11:**
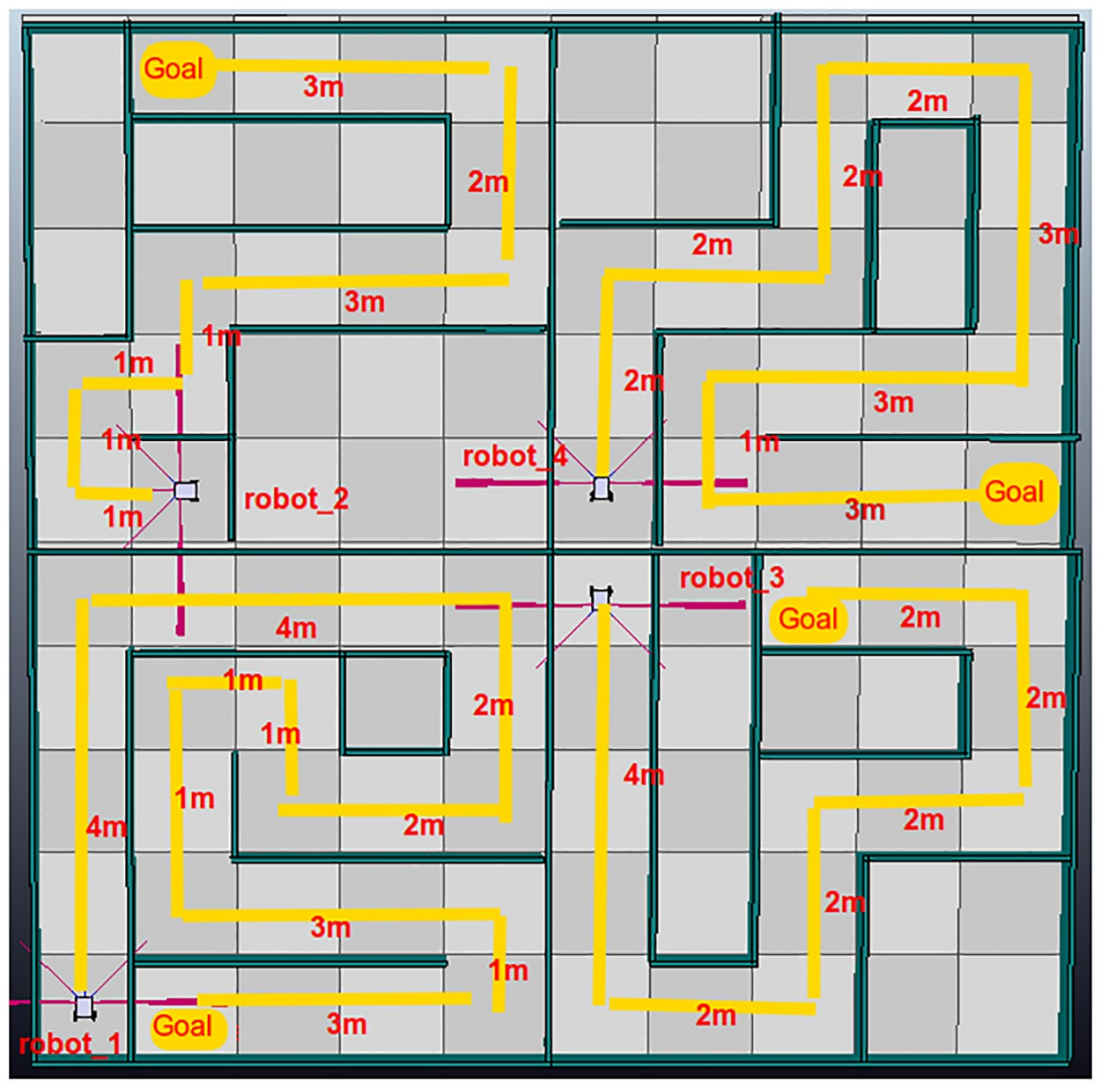
Second test environment created in V-Rep.

**Fig 12:**
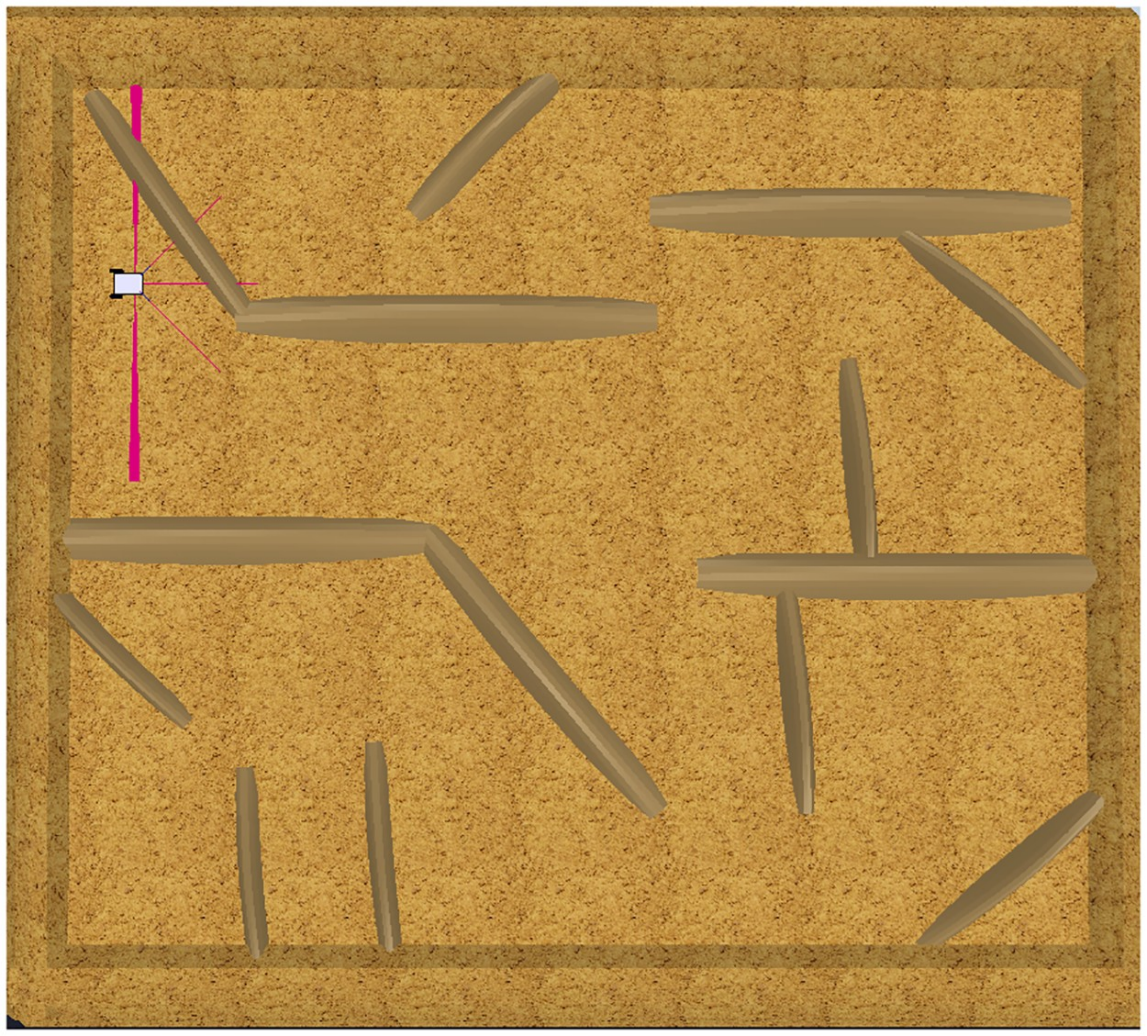
Third test environment created in V-Rep.

The first testing scenario is shown in Fig 10. This is a simple scenario, as it contains a single robotic agent. The second scenario contains 4 robotic agents that must be controlled concurrently to complete the objectives (Fig 11) As this DSL is designed as a script, both scenarios will measure lines of code (LOC), the time to complete the tasks and the ability to create connections to remote hosts to manipulate agents. The third scenario is presented in Fig 12, it has an area of 7*m* × 7.7*m*.

### Results

This section will discuss all the test environments. For this, it presents three subsections to analyze the results of each test environment independently. For the first two test and group of people presented, they were presented with the V-Rep work environment already initialized, a tutorial of the Python framework for the group that had not used it before. Each group was also presented with a PyDSLRep manual. For the third test, only the result map is presented.

#### First environment

**Fig 13:**
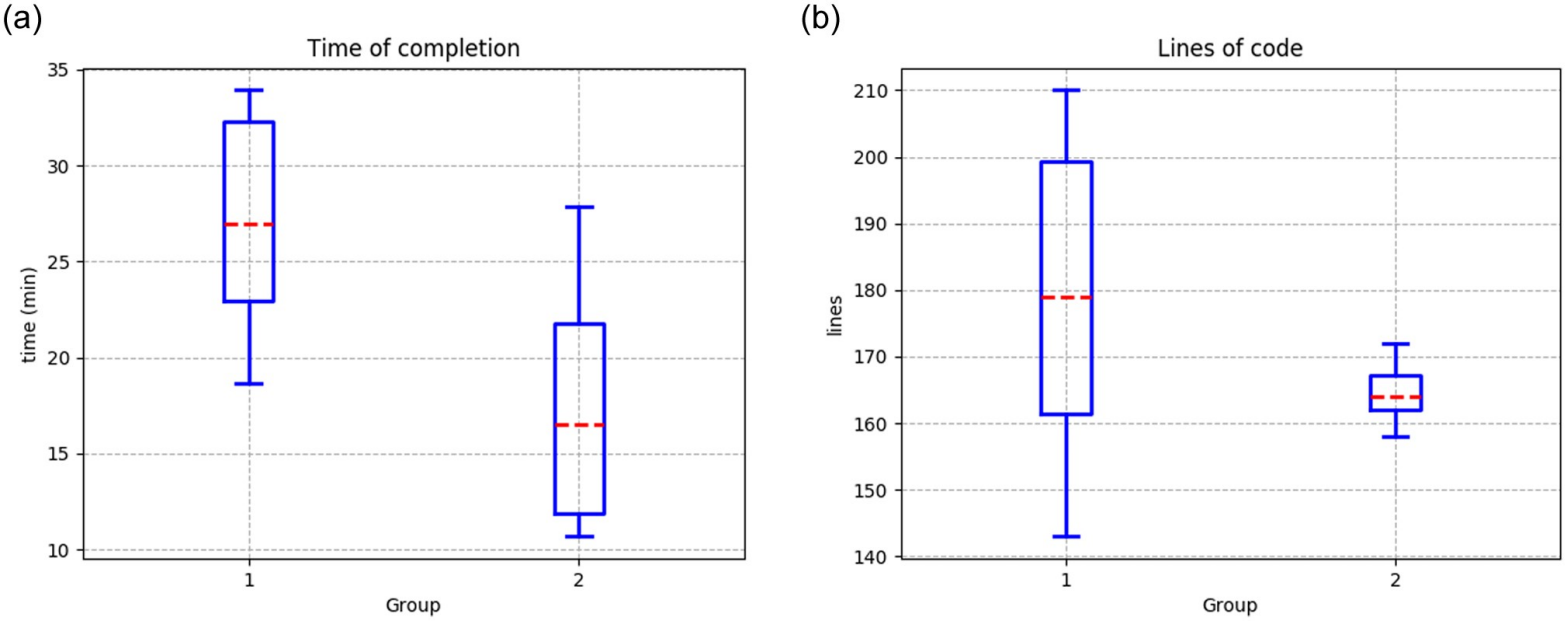
Results from the first environment using the framework in Python (**a**) Time of completion (**b**) Lines of code to fulfill the experiment.

Table 8 presents the results of both groups using the Python framework for robotic agent manipulation in V-Rep. The results show that the first group took an average of 26.94 minutes to solve the problem, with a standard deviation of 5.21 minutes, and the second group showed a shorter average time of 16.53 minutes, with a standard deviation of 6.12 minutes (Fig 13a.)

**Table 8:** Results from the first environment using the python framework.

Subject	Group 1			Group 2		
	LOC	Time (hh:mm:ss)	Remote host	LOC	Time (hh:mm:ss)	Remote host
1	163	00:25:32	yes	162	00:10:48	yes
2	210	00:23:51	no	158	00:27:53	no
3	161	00:21:43	no	162	00:22:47	yes
4	143	00:33:56	no	168	00:13:43	yes
5	207	00:32:51	no	163	00:24:38	yes
6	155	00:33:50	no	172	00:11:69	no
7	175	00:18:39	no	161	00:11:62	yes
8	194	00:30:39	no	162	00:11:51	yes
9	201	00:25:48	yes	165	00:18:52	yes
10	180	00:22:40	yes	168	00:10:40	yes

In the task of connecting with a remote host to conduct the test, 40% of the first group were able to create a successful connection. This result reached 80% in the second group.

Finally, the first group showed an average of 178.9 lines of code, and the second group generated fewer lines of code, with an average of 164.1 (Fig 13b.)

**Fig 14:**
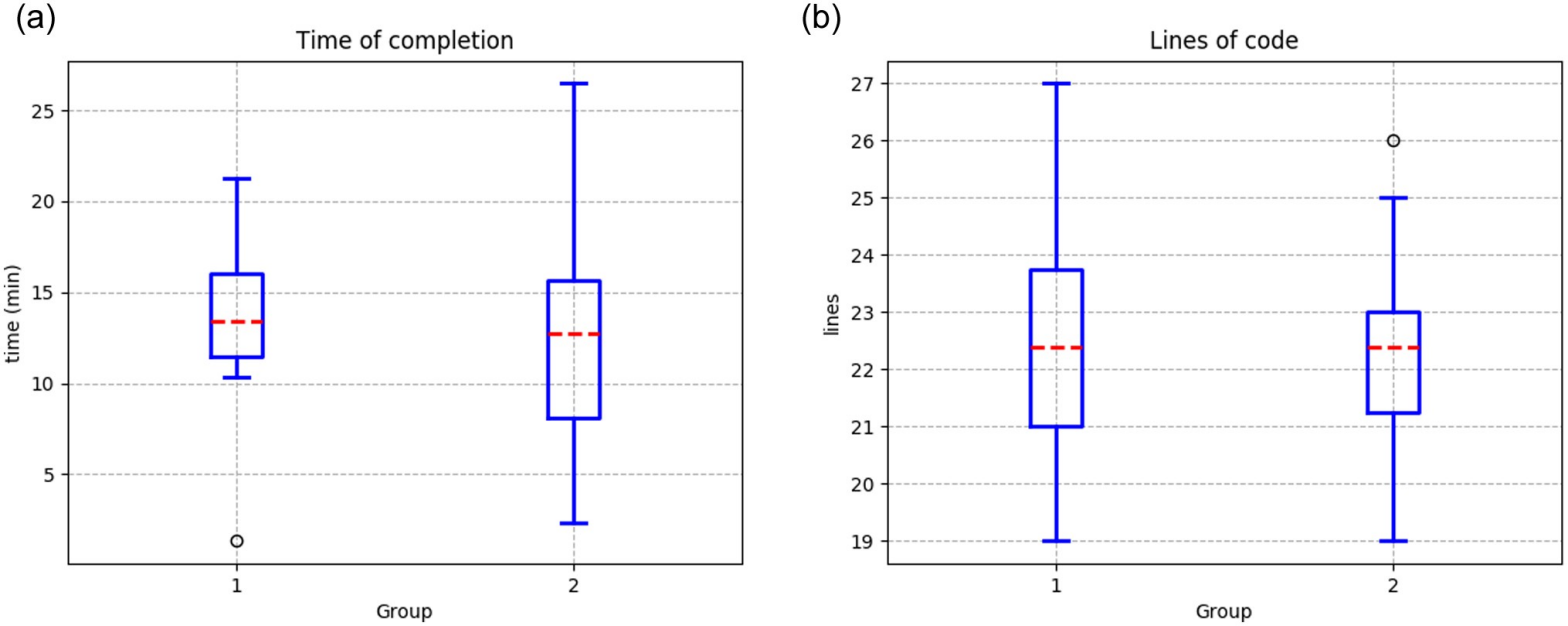
Results from the first environment using PyDSLRep (**a**) Time of completion (**b**) Lines of code to fulfill the experiment.

On the other hand, Table 9 shows the results for both groups using PyDSLRep, in which the first group took an average of 13.39 minutes to solve the problem, with a standard deviation of 5.21 minutes, which is a reduction of 50.29% of the time. Group 2 was also able to reduce the average time to 12.76 minutes with a standard deviation of 6.32 minutes, which is 22.82% as compared to using the Python framework (Fig 14a.)

**Table 9:** Results from the first environment using PyDSLRep.

Subject	Group 1			Group 2		
	LOC	Time (hh:mm:ss)	Remote host	LOC	Time (hh:mm:ss)	Remote host
1	20	00:12:9	yes	21	00:7:19	yes
2	27	00:11:12	yes	23	00:16:3	no
3	23	00:15:14	no	23	00:14:22	yes
4	21	00:19:21	yes	21	00:14:17	yes
5	24	00:21:16	yes	22	00:26:28	no
6	21	00:1:20	no	26	00:8:5	yes
7	24	00:16:15	yes	19	00:8:10	no
8	22	00:10:22	no	25	00:2:18	yes
9	19	00:14:19	yes	22	00:14:18	yes
10	23	00:12:27	yes	22	00:16:14	yes

In the task of connecting with a remote host, the first group increased their connection success to 70%, while the second group decreased by 10%. When the second group was asked about their perception of ease to connect to remote hosts, they replied that they were used to using the Python framework, which made it more difficult to understand the connection with an IP address other than localhost.

Finally, both groups showed a reduction in lines of code used to solve the problem. The first and second group used an average of 22.40 lines of code. For the first group, this represents a reduction of 87.47%, and for the second, it is a reduction of 86.34%, on average (Fig 14b.)

To tell if the differences are significant between the results in the first environment, we apply the ttest. This test is calculated measuring the *t-score* using (7) and the critical value by (8). If the *t-score* is greater than the critical value, the non null hypotheses is approved, that in this case is that PyDSLRep has better results.

$$\begin{array}{c} t\;score=\frac{M_{x}-M_{y}}{\sqrt{\frac{{S_{x}}^{2}}{N_{x}}+\frac{{S_{y}}^{2}}{N_{y}}}} \end{array}$$ (7)

$$\begin{array}{c} cv=\frac{\gamma (\frac{df+1}{2})}{\sqrt{\pi .df}.\gamma (\frac{df}{2}).{\left(1+{\left(1-\alpha \right)}^{2}\right)}^{\left(\frac{df+1}{2}\right)}} \end{array}$$ (8)

Using the ttest, shows that the first group has a significant variation when they develop the task using PyDSLRep with a *t score* = 5.815493492345903 > *cv* = 1.7340636066175354. While the second group doesn’t show a great improvement with a *t* = 1.3774471037866356 < *cv* = 1.7340636066175354.

#### Second environment

The second testing scenario presented to both groups has three differentiating factors as compared to the first scenario. First, they must manage 4 agents, which requires more planning. Second, the movement actions must be executed in parallel. Finally, the distances traveled by the agents vary, which prevents the use of recursive functions.

**Fig 15:**
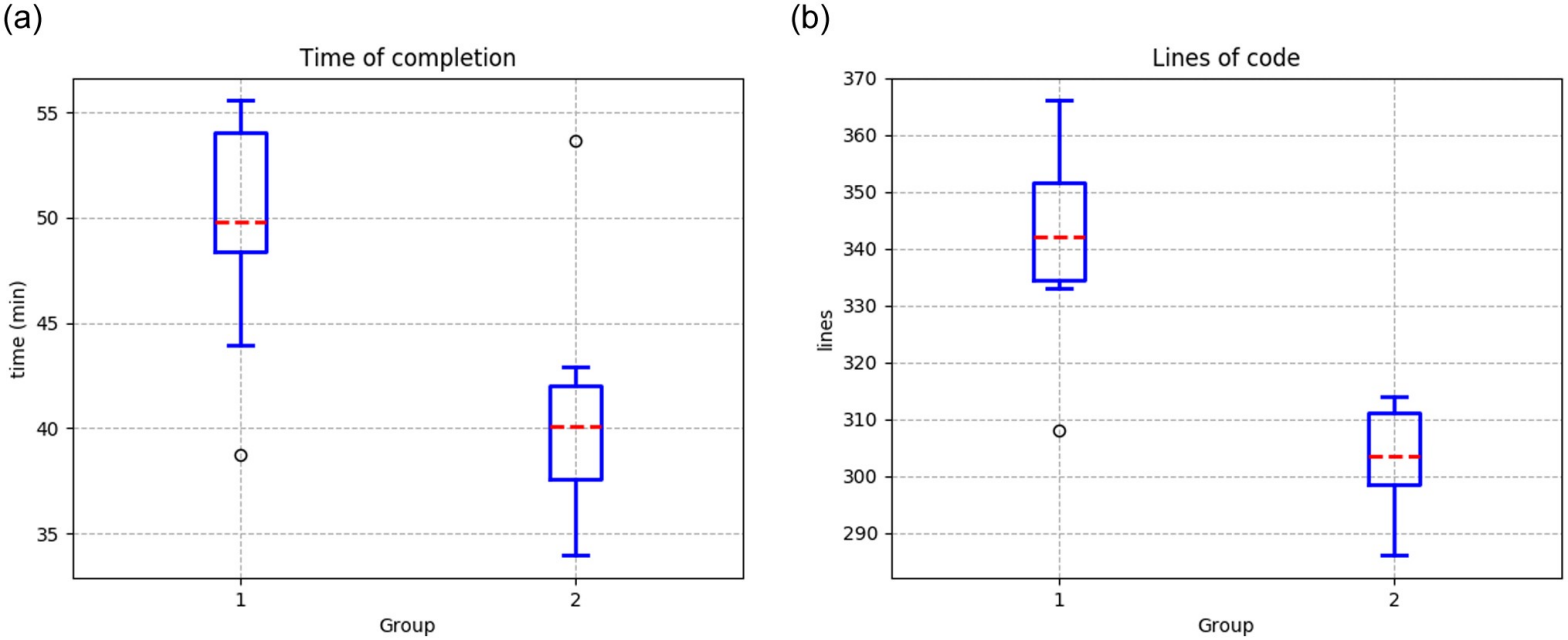
Results from the second environment using the framework in Python (**a**) Time of completion (**b**) Lines of code to fulfill the experiment.

Table 10 presents the results of both groups using the Python framework for manipulating robotic agents in V-REP. The results show that the first group took an average of 49.82 minutes to solve the problem, with a standard deviation of 5.00 minutes. Group 2 required a shorter average time to solve the problem of 40.08 minutes, with a standard deviation of 5.31 minutes (Fig 15a.)

**Table 10:** Results from the second environment using the python framework.

Subject	Group 1			Group 2		
	LOC	Time (hh:mm:ss)	Remote host	LOC	Time (hh:mm:ss)	Remote host
1	333	00:50:54	yes	314	00:42:44	yes
2	353	00:55:34	no	298	00:48:73	no
3	337	00:49:55	no	304	00:37:48	yes
4	334	00:51:51	no	297	00:48:37	yes
5	347	00:47:52	no	286	00:44:66	yes
6	336	00:49:50	no	312	00:34:51	no
7	362	00:54:53	no	314	00:46:53	no
8	366	00:43:56	no	302	00:32:46	yes
9	344	00:38:44	yes	300	00:39:55	yes
10	308	00:54:48	yes	308	00:36:47	yes

In the task of connecting with a remote host, for the first group, 30% were capable of establishing a successful connection. For the second group, this percentage rose to 70%.

Finally, the first group used an average of 342 lines of code, and group 2 generated fewer lines of code, with an average of 303.5 lines (Fig 15b.)

**Fig 16:**
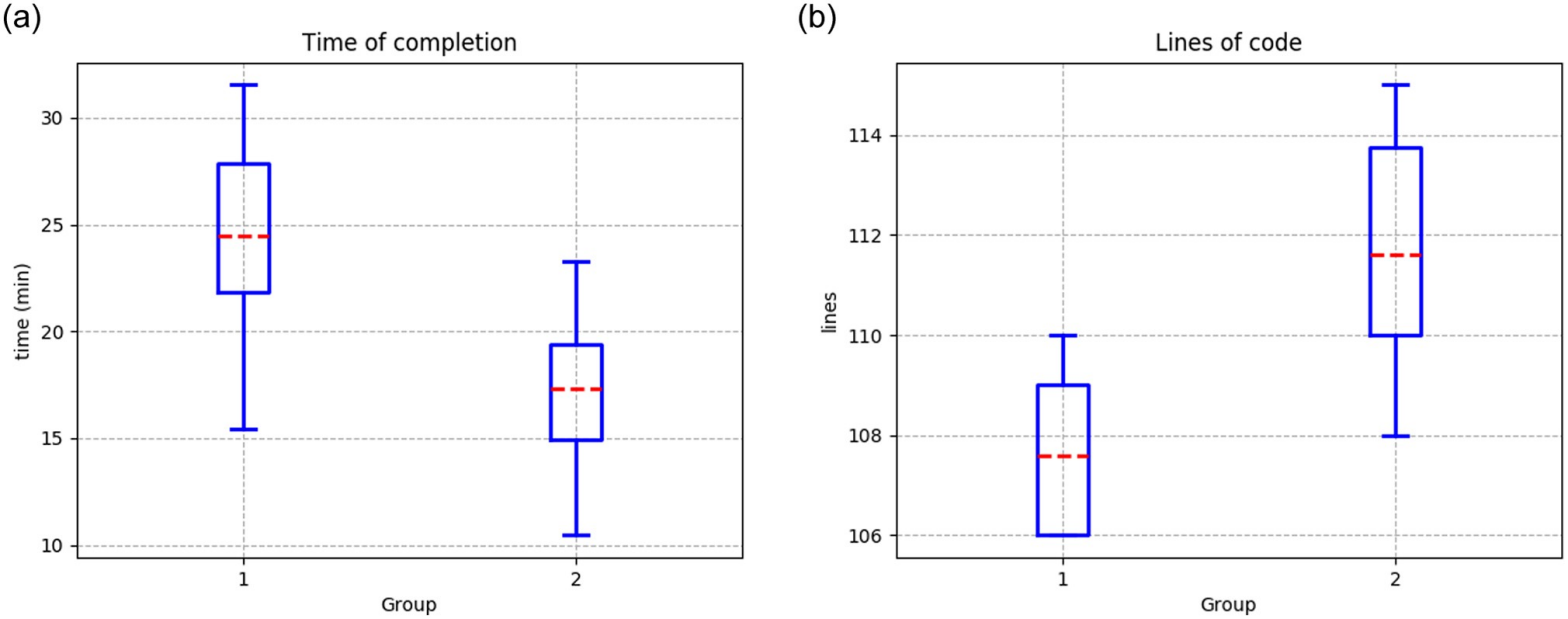
Results from the second environment using PyDSLRep (**a**) Time of completion (**b**) Lines of code to fulfill the experiment.

Table 11 shows the results of both groups using PyDSLRep, in which the first group took an average time to solve the problem of 24.47 minutes, with a standard deviation of 4.73 minutes, which is a 50.89% reduction of time. Group 2 was also able to reduce the average time to 17.30 minutes, with a standard deviation of 3.66 minutes, which is 56.85% of the time required using the Python framework (Fig 16a.)

**Table 11:** Results from the second experiment using PyDSLRep.

Subject	Group 1			Group 2		
	LOC	Time (hh:mm:ss)	Remote host	LOC	Time (hh:mm:ss)	Remote host
1	106	00:19:21	yes	113	00:17:25	yes
2	110	00:23:20	yes	110	00:10:27	yes
3	109	00:25:21	no	114	00:21:26	yes
4	108	00:30:21	yes	112	00:19:23	yes
5	106	00:31:32	yes	109	00:23:18	yes
6	106	00:26:11	yes	114	00:14:25	yes
7	106	00:28:25	yes	108	00:13:19	no
8	106	00:21:20	no	115	00:19:25	yes
9	109	00:15:25	yes	111	00:17:26	yes
10	110	00:23:25	yes	110	00:16:23	yes

In the task of connecting with a remote host, both groups increased the percentage of cases in which they were able to establish a successful connection with the remote host. The first group increased the successful connections to 80%, and the second to 90%.

Finally, both groups showed a reduction in the number of lines of code used to solve the problem. The first group used an average of 107.6 lines of code, which is a reduction of 68.54%. The second group used an average of 110.6 lines of code, which is 63.26% less than using the Python framework (Fig 16b.)

Using the ttest (7, 8), shows that the first group has a significant variation when they develop the task using PyDSLRep with a *t score* = 11.336864645923933 > *cv* = 1.330390943569909. While the second shows a great improvement with a *t* = 9.592773733982149 > *cv* = 8.434164477932882*e* − 09.

#### Third environment

**Fig 17:**
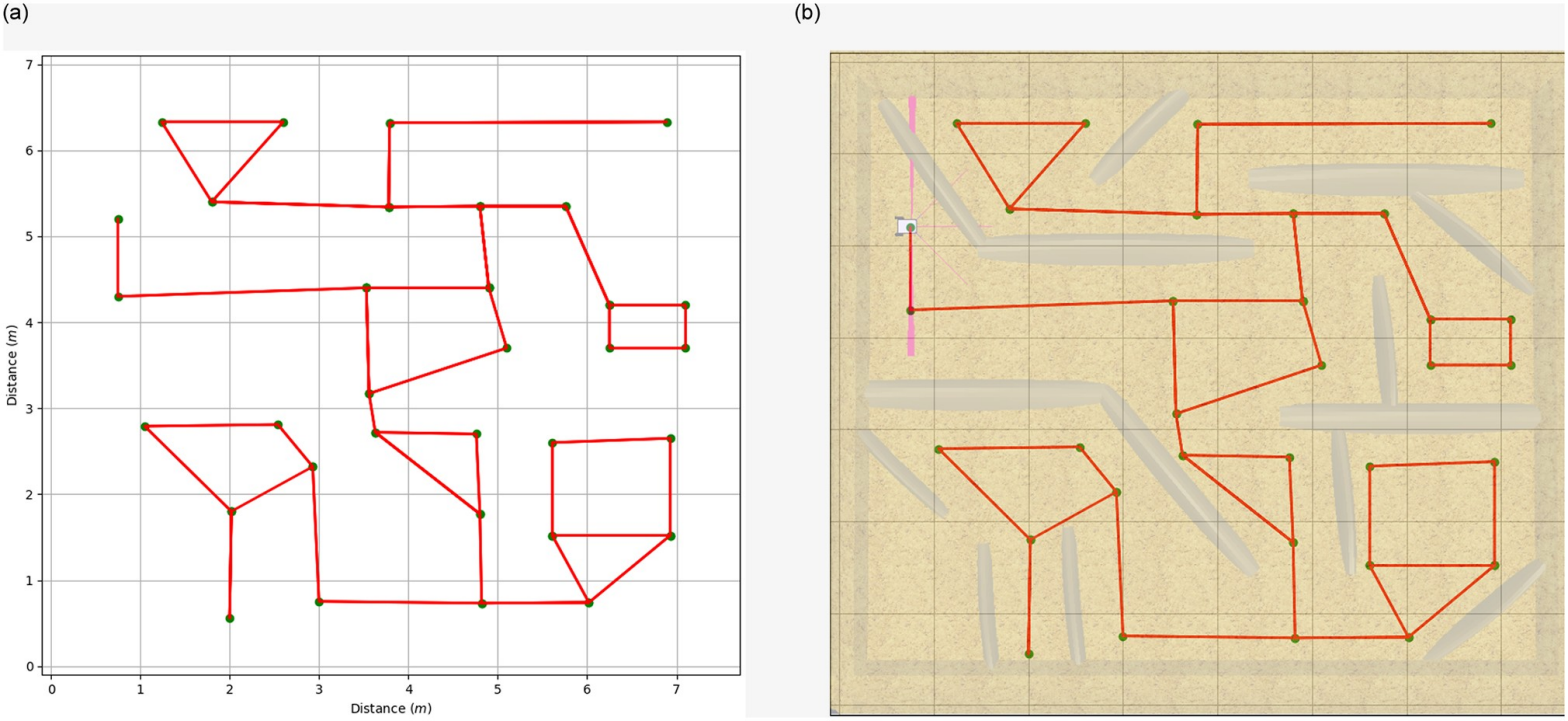
SLAM in PyDSLRep (**a**) topolical map (**b**) overlapped result in the original environment.

The third testing scenario was not presented to the testing groups, this si beacuse PyDSLRep only uses one line of code in the movements declaration to create the map, as it was explained in the Table 7. The map generated by PyDSLRep is presented in the Fig 17.

## Conclusions

DSLs increase the level of abstraction and reduce development time by allowing to generate models and specific domain concepts, enabling developers to use clearer and simpler semantics to write code. This article presents the development and usage of a DSL called PyDSLRep, which enables semantic abstraction for navigation of robotic agents, saving the user from having to generate the dynamic and kinematic models of the robotic agent by applying clear grammar rules used in the specific domain of robotics and applying them to the integrated simulation development environment V-Rep.

Unlike other DSLs, PyDSLRep is not oriented to controlling a specific robotic platform. It also generates plain Python code that can be applied easily to a physical model.

The test performed in this article to validate the usability and effectiveness of PyDSLRep in navigational tasks were done with a total of 20 people who had prior knowledge of the Python programming language. However, only 10 users of this population had the knowledge to use the Python framework to establish communication with robotic agents in V-Rep, allowing the comparison of two groups.

The testing process had three stages. The first stage configured two testing environments with multiple robotic agents, which had to be controlled by using V-Rep’s Python framework. The second stage included the same two development environments, but the control of the agents was done using PyDSLRep. And the last stage was used to perform SLAM in a closed environment.

The results of the first two tests demonstrated that PyDSLRep simplifies the development of navigational routine generation for multiple agents, regardless of having prior experience with the V-REP platform. It reduced the average of generated lines of code by 76.40% and reduced the average development time by 45.22%. And the last test, shows that PyDSLRep could be used for SLAM task, only with a two wheeled differential robot.

Users were also required to connect with a remote host to emulate connection with a physical robotic agent. In this task, PyDSLRep enabled 80% of the users to connect successfully with the remote host, as compared to just 40% of users being able to connect successfully when using the Python framework.

For future work, PyDSLRep’s grammar will be expanded to control different mobile robots and actuators, such as robot arms or Ackerman style robots, and to be capable of reading LIDAR sensors, which would make it capable of generating 3D maps, expanding the language for direct controller generation in physical platforms.
